# Collective decision-making under predator threat is faster in guppy shoals selected for larger telencephalon size

**DOI:** 10.1007/s10071-025-02003-7

**Published:** 2025-10-21

**Authors:** Annika Boussard, Mikaela Ahlkvist, Alberto Corral-López, Stephanie Fong, John Fitzpatrick, Niclas Kolm

**Affiliations:** 1 Stockholm University, Stockholm, Sweden; 2 Uppsala University, Uppsala, Sweden

**Keywords:** Collective cognition, Mosaic brain evolution, Anti-predator behaviour

## Abstract

Avoiding predation is essential for most animals. For group-living species, effective predator avoidance relies on making fast and accurate collective decisions. However, the mechanisms underlying the ability to make adaptive collective decisions and to coordinate movements under predation threat remains unclear. Here, we used guppies artificially selected for divergence in the size of the telencephalon, the main brain region for advanced decision-making in vertebrates, to test the influence of telencephalon size on collective decision-making under predation threat. We measured the latency and accuracy of collective decision-making to avoid a model predator in guppy shoals. In addition, we used high-resolution tracking analysis to assess shoaling dynamics under predator threat between the telencephalon size selection lines. We found that collective decision-making latency was shorter in large telencephalon guppy shoals, indicating that variation in telencephalon size can cause variation in the ability to avoid predation. This result is unlikely to be driven by differences in boldness, as several standard tests suggest that there is no difference in boldness between the telencephalon size selection lines. General aspects of shoaling dynamics did not differ between the telencephalon size selected lines. Our study highlights that rapid mosaic changes in brain region size may be an important mechanism behind social behavioural variation with strong fitness implications.

**Supplementary Information:**

The online version contains supplementary material available at 10.1007/s10071-025-02003-7.

## Introduction

Responding to a predator threat is one of the most important decisions an animal makes, as it can have strong individual fitness consequences. For group-living animals, predator avoidance responses are often the result of collective decision-making. Collective decision-making can be observed when a group of animals turns together and uniformly changes their direction of movement to avoid a predator attack (Couzin 2009; Herbert-Read et al. 2015; Procaccini et al. 2011; Sumpter and Pratt 2008; Sumpter et al. 2008; Ward et al. 2008, 2011). Collective decisions can thus be critical to avoid predator attacks and thereby ensure survival and future reproduction. This has been demonstrated in bird flocks, for instance, where predator success varies with the formation of the escape wave (Procaccini et al. 2011). To date, modeling and empirical investigations provide good accounts of how information on potential threats is transferred between neighbors in large groups and how it enables the group to act as a uniform entity (Dall et al. 2005; Herbert-Read et al. 2015). However, the proximate mechanisms underlying variation in collective decision-making under predator threat in vertebrates remain understudied. A largely unresolved question is how brain morphology influences collective decision-making under predator threat and if evolutionary changes in brain morphology can lead to more effective decision-making. Elucidating this question is important to understand the causes of variation in collective decision-making and the link between group-level properties and individual fitness consequences (Couzin 2009).

At the core of the study of collective decision-making is how animals use information and the behaviours of others to make adaptive decisions (Couzin 2009; Dall et al. 2005; Procaccini et al. 2011; Sumpter et al. 2008; Ward et al. 2008, 2011). Collective decisions are preceded by the spread of information through the group (Couzin 2009; Dall et al. 2005; Sumpter 2008). There can be a divergence in the cognitive processes involved in how consensus in collective decisions can be made. First, when a single individual detects a predator and escapes, near neighbors copy the avoidance behaviour, creating a wave of collective change of direction of movement and thereby escaping the predator attack (Herbert-Read et al. 2015; Procaccini et al. 2011; Ward et al. 2011). Such responses may not require advanced cognitive abilities and enhanced individual cognitive processing of information. Indeed, simple attraction and repulsion forces that do not require advanced cognitive processing have been put forward to underlie collective behaviours in general and also escape waves (e.g., Herbert-Read et al. 2015; Ioannou et al. 2012; Ward et al. 2011; Wood and Ackland 2007). Repulsion-attraction responses refer to basic tendencies of individuals to move away from nearby neighbors (repulsion) or towards others at a distance (attraction), guiding group movement (Couzin and Krause 2003). This is likely the main mechanism involved in collective decision-making for animals living in large fish schools, bird flocks, or giant herds (Couzin 2009; Herbert-Read et al. 2015; Procaccini et al. 2011). Another possibility is that collective decisions are reliant mainly on individual information sampling and cognitive processing. After such individual information is collected and processed, the group coordinates to remain cohesive and make an adaptive collective decision (Sagot et al. 2023). This indicates that collective decision-making to avoid predators can be a social decision, which may require different and more advanced cognitive abilities in contrast to the simpler attraction-repulsion forces (Sagot et al. 2023; Sueur et al. 2011). This is more likely the case for animals living in smaller groups than in groups of hundreds to thousands of individuals.

Social group living has been associated with cognitive challenges such as the need to recognize and remember group members, navigate complex social hierarchies, manage cooperation and competition, and respond to social cues and conflicts (Ashton et al. 2018; Shultz and Dunbar 2007; Street et al. 2017). Increased investment into brain size that generates enhanced cognitive abilities has been suggested to evolve to cope with such challenges (DeCasien and Higham 2019; Street et al. 2017). One brain region that has received attention in the context of social cognition is the telencephalon (Fischer et al. 2015; Triki et al. 2019, 2020). The telencephalon is the main brain region responsible for processing sensory information to generate a behavioural response (Butler and Hodos 2005). As such, the telencephalon is the primary region for higher-order integrative brain functions, such as learning, working memory, and spatial navigation (Broglio et al. 2003, 2005; Butler and Hodos 2005; Guillemot 2005; Shimizu et al. 2010; Triki et al. 2022, 2023). To avoid a predator attack, an animal must detect the predator through sensory cues, assess attack motivation, and decide on an appropriate behavioural response. Given the function of the telencephalon, an increased investment in neural tissue in this brain region may facilitate the processing of visual sensory information on a potential threat and generate a faster and more accurate behavioural response to avoid a predator attack, especially if the decision to escape is initially based on individual information processing, and group members then organize themselves to coordinate a suitable response. Whether a larger telencephalon facilitates cognitive processing during collective decision-making remains unknown. Empirical tests investigating whether telencephalon size influences collective decisions to successfully avoid predation are paramount to solving the issue.

Here we use predator-naïve guppies (*Poecilia reticulata*, W. Peters 1859) from an artificial selection experiment for small and large relative telencephalon size. After five generations of selection, a difference of approximately 10% in telencephalon volume has been established between up- and down-selected lines (Fong et al. 2021; Triki et al. 2023). We ask whether collective decision-making under predator threat differs between single-sex guppy shoals from the telencephalon size selection lines. Using established protocols (Kotrschal et al. 2018, 2020; Ward et al. 2008, 2011), including high-resolution trajectory data, we quantify two aspects of social dynamics: (1) collective decision-making, which involve the group coordinating responses to external threats, and (2) shoaling structure, which reflect the spatial organization and social cohesion within the group. While collective decision-making is one component of social dynamics, shoaling structure represents another distinct facet of social interaction. We hypothesize that shoals with larger telencephalon size will perform better in collective decision-making and exhibit more cohesive shoaling dynamics compared to those with smaller telencephalon size. Such a result would suggest that variation in telencephalon size influences multiple components of social dynamics, possibly through enhanced cognitive abilities. Alternatively, if differences between selection lines are observed only in decision-making but not shoaling structure, this would indicate that only specific aspects of collective behaviour depend on telencephalon size.

## Materials and methods

- Model system and husbandry.

We conducted the study in the fresh water aquarium facilities at Stockholm University between March and April 2021. We used laboratory bred descendants to guppies caught in 1998 in high-predation areas in the Quare River in Trinidad. The artificial selection procedure was performed by Fong et al. (2021) for generation one to four and thereafter by a lab technician. Extensive details on the selection procedure can be found in Fong et al. (2021). Briefly, the selection procedure started with three breeding stocks (hereafter replicates) that were up- and down-selected for relative telencephalon size (i.e., three up-selected lines and three down-selected lines in total). The offspring of 75 breeding pairs per replicate, i.e., 225, with the 20% smallest vs. 20% largest relative telencephalon volume (relative to the rest of the brain) as the F_1_ generation were used. The 225 breeding pairs were euthanized and their brains dissected out to assess telencephalon size. After five generations of this selection procedure, the small and large telencephalon size selection lines differed approximately 10% in telencephalon volume in both sexes (Fong et al. 2021; Triki et al. 2023).

We housed sexually mature fish in 7 L and 40 L stock tanks in groups of 8–9 females and 40 males, sorted by sex, replicate and selection lines. Sexual maturation was assessed as the first onset of gonopodium development in males and gravid spot in females, which occurs around two-three months age. Fish were kept in these closed, recirculating aquarium systems for eight months prior to being moved to holding tanks (see below). We kept the laboratory at a 12:12 dark: light scheme and with 25 ± 1 °C water temperature. All tanks were enriched with two cm gravel, biological filter, snails (*Planorbis sp*) to reduce biological waste products, java moss (*Taxiphyllum sp.*) and/or artificial plants. We fed fish daily with flake food and *Artemia* hatchlings interchangeably.

For this study, we used guppy shoals that had previously been used in an open field test (Boussard et al. 2024), but unexperienced with predators. A total of 968 sexually mature males and females were collected by AB and MA from stock tanks one month prior to the experiments, equally distributed among the sexes, replicates and selection lines. These shoals were housed in single-sex shoals of eight individuals in separate 7 L transparent holding tanks throughout the experiment (i.e., 121 shoals in total, 60 small and 61 large telencephalon size guppy shoals). Guppy shoals were never mixed between holding tanks prior to or during testing, i.e., the guppy shoals were always tested in familiar shoals. To minimize observer bias, a person unrelated to the experiment labelled holding tanks with running numbers. Prior to testing, we transferred the fish shoals from their holding tanks to the experimental arenas in a cup with water to minimize stress. To ensure that conspecific chemical cues remained relatively constant we changed over half of the water between trials. Each shoal was tested only once, and the order of testing was randomized across selection lines, sexes, and replicates to avoid systematic timing effects. We tested fish between 9.00 and 18.00 on week days.

(b) Collective decision-making.

**Fig. 1:**
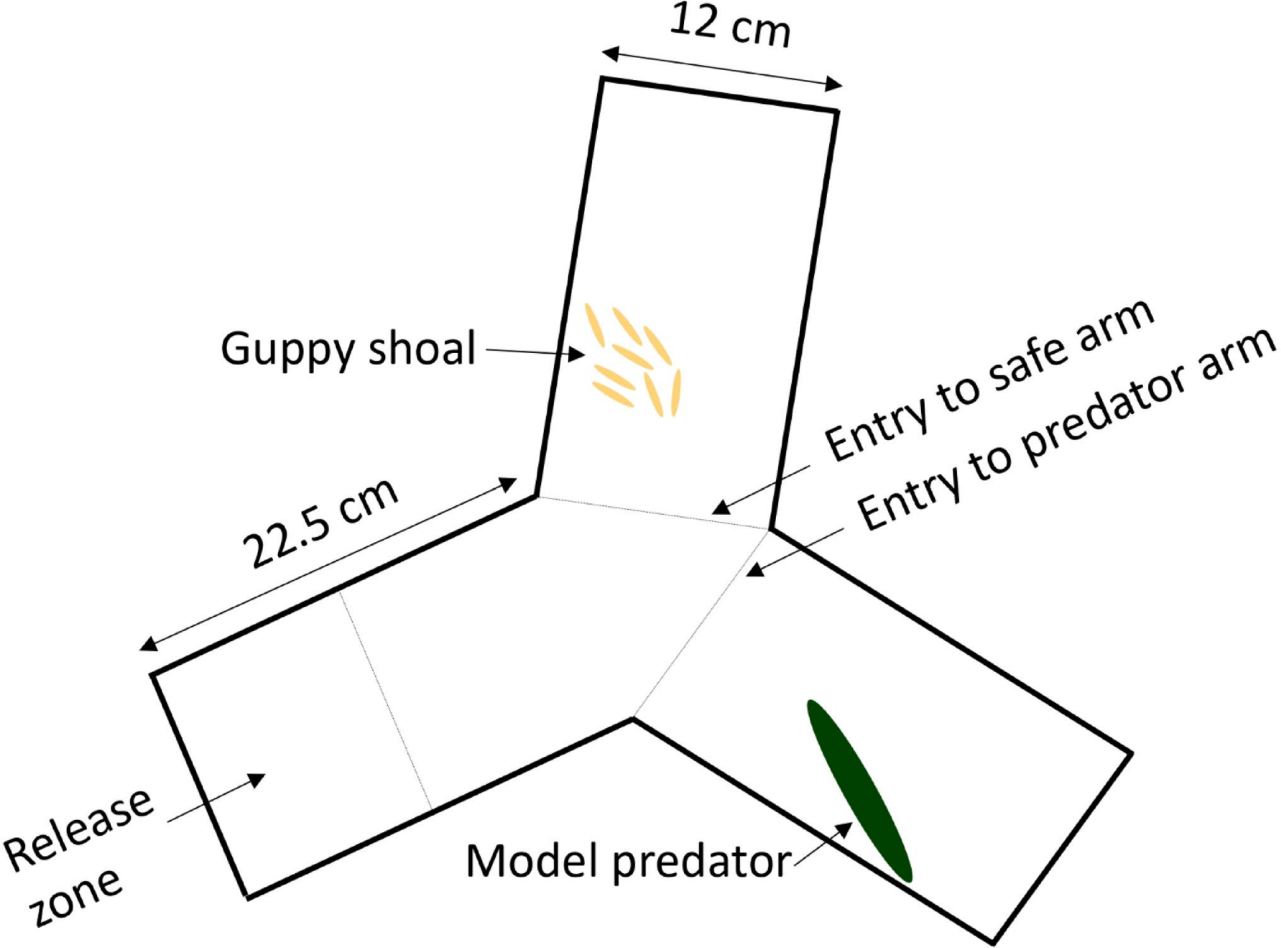
Experimental apparatus

We investigated collective decision-making during predator threat separately in male and female shoals for ecological and practical reasons. Female guppies tend to form more stable shoals, while males tend to shift frequently between shoals (Croft et al. 2003). In addition, mixed sex shoals likely disrupt the behaviours of interest here due to the potential for mating behaviours to interact with anti-predator or shoaling behaviours (Croft et al. 2003; Houde 1997). We assessed 121 guppy shoals from the small and large telencephalon size selection lines in a white symmetrical y-maze (small telencephalon size selected: *N* = 60, 30 female- and 30 male shoals; large telencephalon size selected: *N* = 61, 31 female- and 30 male shoals). The two copies of these mazes were 12 cm wide and 22.5 cm long per arm, with approximately four cm water depth (Fig. 1). The length of the mazes ensured that the majority of guppies within the shoal detected the predator model approximately at the same time. This would ensure that all guppies individually sampled and processes sensory information and thereafter coordinated with the group to make a collective decision. In one arm of each maze, we placed a stationary model predator 0.5 cm from the bottom by gluing them on a circular shaped magnet with 0.5 cm in diameter. The model predators were fully covered by water. As model predators, we used two 12 × 3 cm long fishing lures, custom-painted to resemble the pike cichlid (*Crenicichla frenata*). Pike cichlids are natural predators of the guppy (Houde 1997). These model predators have successfully induced different aversive behaviour than towards a novel object, e.g., differences in number of predator inspections, distance and activity around the object, in guppies in other studies (van der Bijl et al. 2015; Corral-López et al. 2020, 2023). To account for any lateralization bias, half of the shoals had the model predator in the right-hand side arm from the release arm, and vice versa for the other half of the shoals. We transferred the shoals into a transparent PVC cylinder in the release zone (Fig. 1). The cylinder was 12 cm in diameter, matching the size of the y-maze. After 20 min acclimatization we raised the cylinder and the shoal was released. The same procedure was applied to both female and male shoals. All trials were filmed with Point Grey Grasshopper 3 cameras (FLIR Systems; resolution, 2048 pixels by 2048 pixels; frame rate, 25 Hz) that were placed above the arenas. We started the recording when we raised the cylinder and continued to record for two minutes with 30 frames per second, i.e., 30 data points/sec on individual positions. We chose the time limit based on anti-predator behaviour during previous experiments with model predators (van der Bijl et al. 2015). AB and MA collected the data from watching live videos, or the video recordings when necessary. We defined three zones for the collective decision-making analysis. These zones were not actual barriers, only imaginary barriers to facilitate measurements. We designated the arm that contained the model predator as the “predator arm”, and the other arm as the “safe arm” (Fig. 1). We measured two aspects of collective decision-making: (1) latency (s), following the protocol established by Ward et al. (2011) latency was defined as the time from the shoal was released until the majority of fish (i.e., five out of eight) had made their way to entry the safe arm, and (2) accuracy, defined as the number of fish per shoal that initially chose the safe arm vs. the predator arm (i.e., we measured if their first choice were to enter the safe or the predator arm after leaving the release zone). Accuracy in collective contexts is usually referred to the proportion of the group, or the number of individuals within an animal group, that is making a certain decision (Franks et al. 2003; Ward et al. 2008, 2011). Latency and accuracy are important aspects of collective decision-making in the context of avoiding a predator (Procaccini et al. 2011; Sumpter et al. 2008; Ward et al. 2008, 2011), especially since latency and accuracy are sometimes in opposition (Franks et al. 2003). Accurate collective decisions decrease the risk of the group splitting apart and reduces the ability of predators to target individuals (Ioannou et al. 2012; Wood and Ackland 2007). Making fast collective decisions can reduce the time the animal group is exposed to risk and a potential predator attack. Animal groups should optimize accuracy and latency for collective decision-making to be adaptive when threatened by a predator. Such optimization could require advanced cognitive processing.

(c) Shoaling dynamics.

We investigated shoaling dynamics under predator threat in 120 guppy shoals from the small and large telencephalon size selection lines. This was performed simultaneously as the collective decision-making assay. We tracked the individual movement of all fish in the shoal from the video recording with idTracker (Pérez-Escudero et al. 2014). We used the tracking data to calculate main sub-group size, alignment, distance to nearest neighbor (henceforth attraction) and speed in MATLAB 2020 following Kotrschal et al. (2018, 2020). We chose these group level variables as they have been shown to characterize the structure and dynamics of guppy shoals well (Herbert-Read et al. 2017; Sumpter et al. 2018). Main sub-group size was calculated as the average number of fish within the largest sub-group, where the distance between individuals was less than 100 mm (representing approximately 3.5 body lengths). We followed the optimization process outlined in Szorkovszky et al. (2018) and calculated the median global alignment per guppy shoal. The global alignment represents the angular alignment among all individuals in the arena throughout all frames. A high alignment score indicates that all fish are oriented in the same direction, whereas a low alignment score indicate that all fish are oriented in different directions. We calculated attraction as the median distance in mm to the nearest neighbor across all frames. Speed [mm*s^−1^] was determined as the median speed for all shoal members, achieved by calculating the first derivatives of the x and y time series and applying a smoothing process using a third-order Savitzky-Golay filter. We estimated body size from idTracker based on calculation of the mean number of pixels per shoal. To ensure robust results, we disregarded tracking data with less than 70% completed tracks from further analyses. This happened in two shoals from the small- and one shoal from the large-telencephalon size selection lines (reducing the final sample size to: *N* = 117). For full details on the calculations, please see Kotrschal et al. (2018) and Sumpter et al. (2018).

(d) Statistical analyses.

We ran all analyses and generated all figures in the open-access software R studio (v 4.3.2, http://R-project.org/). All raw data and R code are available for editors and reviewers at https://figshare.com/s/c43d3666a842ee066b85 and will be publicly available upon acceptance.

We present the effects of the covariates replicate, speed when controlling for activity and body size for respective models in the supplementary material, Table S1.

The neural response to artificial selection on relative telencephalon size has during five generations shown similar patterns between males and females (Fong et al. 2021). Hence, we would not expect sex-specific differences in behaviour between the selection lines (Boussard et al. 2023, 2024). Sex effects were therefore not explored here. Also, adding sex to the models resulted in qualitatively similar results. For transparency, we present additional models including sex in the supplementary material, Table S2 and S3.

(i) Collective decision-making.

To determine whether relative telencephalon size significantly affected collective decision-making under predator threat, we fitted separate models for the response variables (latency and accuracy) as a function of the explanatory variable telencephalon size_(small, large)_. Replicate was included as a three-level covariate to account for potential differences between the replicated selection lines, but was only retained if the effect was significant (*p* < 0.05). For the model examining latency, we performed a Cox regression by using the *coxph* function in the *survival* package (Therneau 2023). To check that the model met the proportional hazard assumption, we used the *cox.zph.* function. The Cox proportional hazards model was chosen because it provides a robust framework for analyzing time-to-event data while simultaneously accounting for both the timing and the occurrence of choices. The Cox model incorporates the full distribution of decision times and avoid imposing assumptions about the underlying distribution of decision latencies. Test statistics and p-values were obtained by using the *summary* function in the *survival* package (Therneau 2023).

For the model examining collective decision-making accuracy, we performed a generalized linear model (GLM) with logit link functions as implemented in the *lmer* functions in the *lme4* package (Bates et al. 2014). The response variable was modelled as proportion data (i.e., “safe arm, predator arm” by using the *cbind* function) under a binomial assumption. By dividing the sum of squared Pearson residuals with the residual degrees of freedom, we found no evidence for overdispersion (ratio = 2.54). Test statistics and p-values were obtained by using the ANOVA function specifying Type III Wald chi-square tests in the *car* package (Fox and Weisberg 2019).

(ii) Shoaling dynamics.

To determine whether relative telencephalon size selection line significantly affected shoaling dynamics under predator threat, we fitted four separate sets of linear models (LM) by using the *lm* functions in the *stats* package. We modelled the response variables (sub-group size, alignment score, attraction and speed respectively) as a function of the explanatory variable telencephalon size_(small, large)_. Since alignment interacts with swimming speed (Kent et al. 2019), we included speed as a continuous covariate in the model examining alignment score. Replicate was included as a three-level covariate to account for any potential difference between the replicated selection lines, but was only retained if the effect was significant (*p* < 0.05). Mean body size (standardized by mean centering and divided by 1 standard deviation) was incorporated as a covariate in all models. The response variables were power transformed to meet the model assumptions when necessary. The assumptions for normality and equality of variances were confirmed by visual inspection of the residuals. Test statistics and p-values were obtained by using the ANOVA function specifying Type III Wald chi-square tests in the *car* package (Fox and Weisberg 2019).

## Results

- Collective decision-making.

**Fig. 2:**
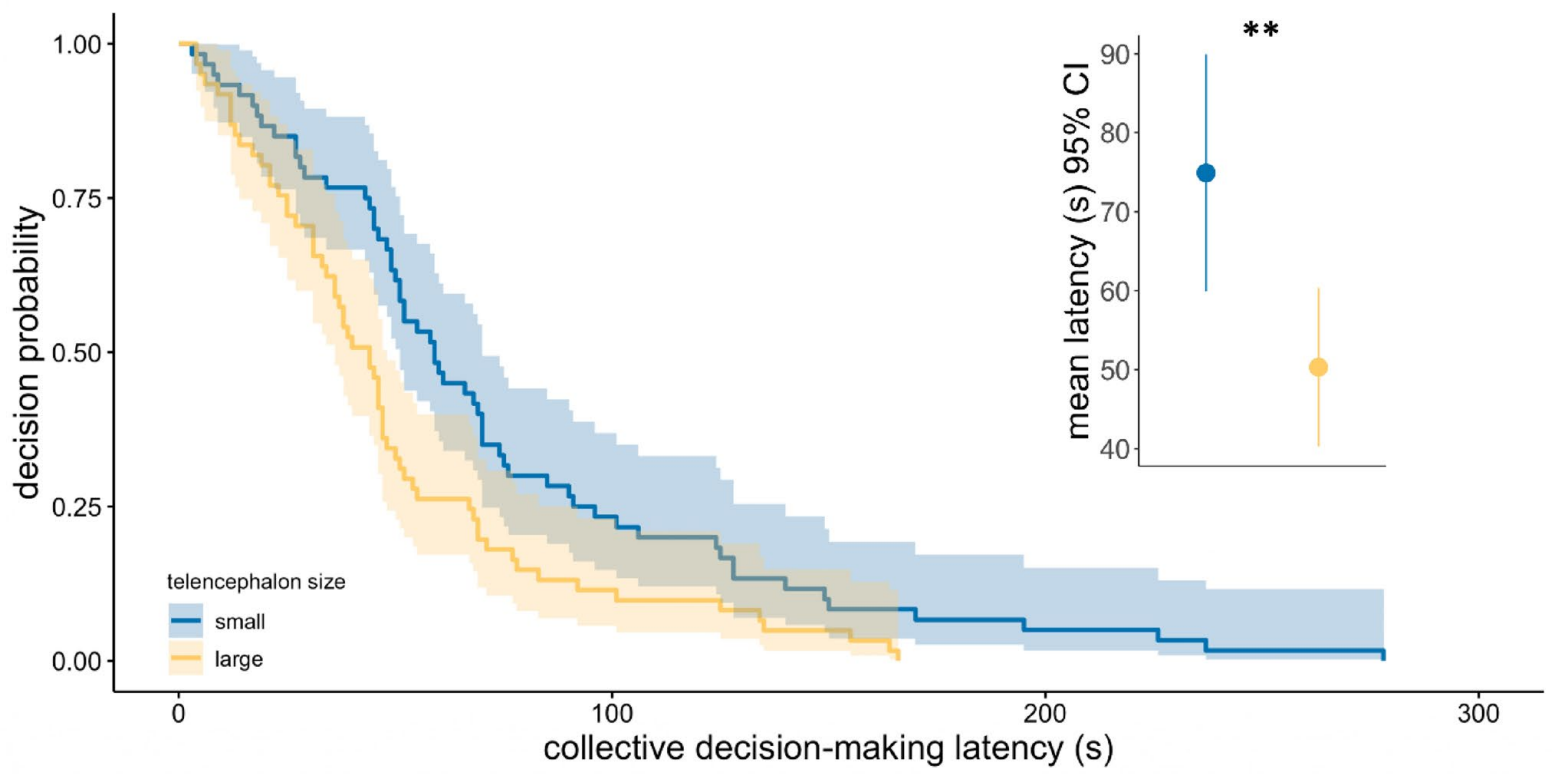
Collective decision-making to avoid a predator. Collective decision-making latency (s) in single-sex guppy shoals from small (blue) and large (yellow) relative telencephalon size selection lines. Shown is the decision probability of the majority (five out of eight) of guppy shoals choosing the safe arm in a y-maze with a model predator at different time points. The solid lines represent the Cox regression model predictions, accompanied by a 95% confidence interval. The model shows that guppy shoals from the large telencephalon size selection lines were significantly faster than guppy shoals from the small telencephalon size selection lines (*p* = 0.005). Inserted in the upper right corner are boxplots accompanied with data point per shoal. Horizontal lines indicate medians, boxes indicate the interquartile range, and whiskers indicate all points within 1.5 times the interquartile range. Data from 121 shoals

Guppies from the large telencephalon size shoals had on average 32.8% shorter latencies to reach the safe arm than guppies from the small telencephalon size shoals (mean (s) ± s.e.: 50.31 ± 5.02_large telencephalon_ vs. 74.92 ± 7.50_small telencephalon_). The model revealed that collective decision-making latency was significantly shorter in the large telencephalon size guppy shoals (Cox regression; HR ± 95% CI = 1.70 (1.17, 2.45), *p* = 0.005; Fig. 2). To ensure that these results were not caused by potential differences in boldness, we performed an additional assessment of time in seconds to leave the release zone in the start arm. Time to leave the release zone was not dependent on telencephalon size (GLM; χ^2^_1_ = 1.35, *p* = 0.24).

We found that collective decision-making accuracy was not dependent of telencephalon size (GLM; χ^2^_1_ = 2.07, *p* = 0.15). The majority of fish avoided the predator arm and choose the safe arm (mean s ± s.e.: 6.45 ± 0.16_safe arm_ vs. 1.52 ± 0.16_predator arm_).

(b) Shoaling structure and dynamics.

First, we asked if shoal structure differed between the telencephalon size selection lines. We found that sexes from the small and large telencephalon size selected lines formed main sub-group of similar size (average ± s.e.: large telencephalon females 6.3 ± 0.2, small telencephalon females, 6.0 ± 0.2, large telencephalon males 6.7 ± 0.2, and small telencephalon males 6.6 ± 0.2). Indeed, main sub-group size was not significantly different between the small and large telencephalon size shoals (LM; estimate ± s.e. = 1.30 ± 1.19, F_1,112_ = 1.20, *p* = 0.28).

**Fig. 3:**
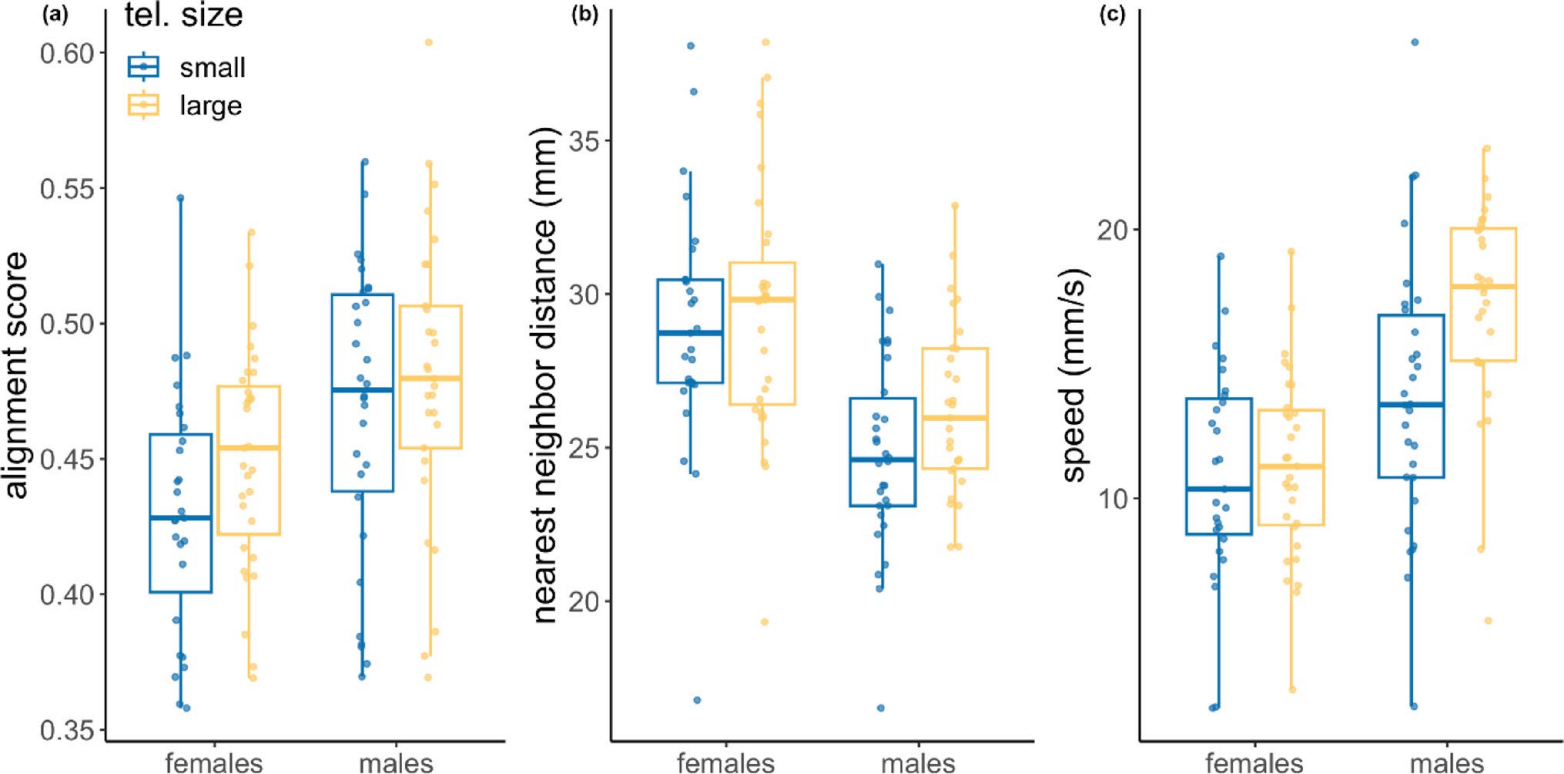
Shoaling dynamics in guppy shoals under predator threat. Boxplots of (**a**) alignment score, (**b**) attraction to nearest neighbor, and (**c**) speed for female and male guppy shoals artificially selected for small (blue) and large (yellow) relative telencephalon (tel.) size assayed in a y-maze allocated with a model predator. Blue and yellow markers show median data points per shoal. Horizontal lines indicate medians, boxes indicate the interquartile range, and whiskers indicate all points within 1.5 times the interquartile range. Data from 117 shoals

Second, we asked whether shoaling dynamics under predator threat differed between guppy shoals from the small and large telencephalon size selection lines. We found no significant effect of telencephalon size on alignment score (LM; estimate ± s.e. = 0.007 ± 0.01, F_1,113_ = 0.56, *p* = 0.45; Fig. 3a). This means that guppies from the small and large telencephalon size selection lines were equally aligned when swimming in a group of eight fish under predator threat in a y-maze. We found no significant effect of telencephalon size selection line on attraction (LM; estimate ± s.e. = 0.78 ± 0.66, F_1,114_ = 1.38, *p* = 0.24; Fig. 3b), which suggests that guppies were equally attracted to their nearest shoal neighbors regardless of telencephalon size. Consistent with our finding that larger telencephalon size shoals were faster to enter the safe arm of the y-maze, we found that the swimming speed (measured as the median speed for all shoal members during two minutes, see methods) was higher in large telencephalon size shoals (LM; estimate ± s.e. = 1.70 ± 0.64, F_1,112_ = 7.14, *p* = 0.01; Fig. 3c).

## Discussion

This study represents the first examination of the impact of experimentally induced differences in telencephalon size on a fitness related behaviour. We found that during cognitively demanding processes a larger telencephalon is important, resulting in shorter collective decision-making latency. However, we found that collective decision-making accuracy was generally high among the guppy shoals, and independent of telencephalon size. Shoaling dynamics under predator threat were also independent of telencephalon size, consistent with the demonstrated simple neighbor interaction rules governing collective movements. Overall, our results suggest that telencephalon size plays a role in more complex cognitive processes most likely involved in predator avoidance behaviour, rather than impacting the more fundamental aspects of cognition underlying shoaling dynamics.

Guppy shoals from the large telencephalon size selection lines were faster to enter the safe arm and avoid the model predator in the y-maze. This result suggests that telencephalon size has a positive effect on collective decision-making speed under predator threat. This could be explained by differences between the telencephalon selection lines in (i) cognitive ability, or (ii) trade offs between speed and accuracy. First, detecting and assessing a predator threat and making a decision to change direction to collectively escape predators may require enhanced cognitive processing, as proposed by for instance Kotrschal et al. (2015, 2017), van der Bijl et al. (2015) and Møller and Erritzøe (2014). Hence, it is likely that the faster collective decision-making in the large telencephalon size guppy shoals was driven by faster sensory information processing and enhanced cognitive abilities in the large telencephalon selection lines. Second, it is important to point out that collective decision-making speed can trade off with accuracy (Burns 2005), also in a collective motion context (Sumpter and Pratt 2008; Franks et al. 2003). However, we found no such pattern in our data. Although collective decision-making was faster in guppy shoals from the large telencephalon size selection lines, accuracy was similar between the selection lines. We therefore think that it is unlikely that a trade off between speed and accuracy in the large telencephalon selection guppy shoals have influenced our results. Taken together, our results suggest that when faced with a predator threat, guppies with a larger telencephalon size exhibit faster cognitive processing, allowing them to assess the situation more rapidly and to make faster collective decisions. By making swift and coordinated responses, such as changing direction or seeking safety, the shoal can increase its chances to avoid a predator and reducing predator success. These cognitive advantages may allow shoals formed by individuals with large telencephalon size to make faster collective decision-making, ultimately enhancing individual survival and fitness by collectively successfully avoid predation. Future experiments on these selection lines will focus on disentangling the exact mechanism behind these results.

Boldness, fast decision-making, and exploratory behavior are often linked because bolder individuals tend to take greater risks and engage more readily with novel environments, which can lead to quicker decisions and increased exploration (Cote et al. 2011; Fraser et al. 2001; Mamuneas et al. 2015). In this framework, differences in boldness could potentially explain faster collective decision-making in groups. However, our comprehensive assessments provide strong evidence against boldness differences driving the observed effects. Previous assays using three standard boldness tests, the emergence test, open field test, and novel object test, revealed no differences between the telencephalon size selection lines (Fong 2020). Similarly, Boussard et al. (2024) found no differences in distance to arena center, a commonly used proxy for boldness (Burns 2008; Maximino et al. 2010), among the same guppy shoals used here. Furthermore, we observed no differences in time to leave the release zone between the groups. Taken together, these data strongly suggest that differences in boldness do not underlie the faster collective decision-making exhibited by the large telencephalon guppy shoals.

Another possibility, which we also find unlikely, is that differences in other brain regions might influence the results. Previously, we have not found any changes in other brain regions in the telencephalon size selection lines (Fong et al. 2021). However, it was recently detected that males from the fifth generation of artificial selection exhibited smaller optic tecta (De Meester et al. 2025; Triki et al. 2023). The optic tectum functions to receive and process visual sensory information and distribute it to other regions of the brain (Butler and Hodos 2005; Suzuki et al. 2019). Consequently, the optic tectum enables animals to coordinate and orient body movements toward the source of sensory inputs (Butler and Hodos 2005; Suzuki et al. 2019). Furthermore, optic tectum size has been positively associated with performance in visual cognitive tasks (Triki et al. 2022). However, our findings contradict with this prediction. Although the guppy shoals selected for a larger telencephalon size in this study may possess smaller optic tecta, they exhibited faster collective decision-making compared to guppy shoals with smaller telencephalon sizes. Therefore, we conclude that potential differences in optic tectum size have not influenced the results.

We found that the number of individuals per shoal that initially chose the safe arm vs. the predator arm was similar between the telencephalon size selection lines. Hence, our result suggests that collective decision-making accuracy was similar between the selection lines. The high accuracy among the guppy shoals is most likely explained by the ease of the task in this set-up, i.e., avoid a predator or not. An easy decision, such as avoiding to swim directly towards a predator, may not require enhanced cognitive processing generated by a larger telencephalon. Instead, such cognitive abilities are most likely aspects of more fundamental cognitive processes. In recent years it has become increasingly clear that fundamental cognitive processes do not always require additional neural tissue invested into the telencephalon (Triki et al. 2022, 2023) or the whole brain (Buechel et al. 2018). While a larger telencephalon or larger whole brain are advantageous in more cognitively demanding tasks (Buechel et al. 2018; Triki et al. 2022, 2023). Our results are in strong agreement with these previous results, since collective decision-making accuracy, was overall very high among the guppy shoals of both selection lines. Whereas making accurate collective decisions fast are presumably more cognitively demanding, and a larger telencephalon is advantageous in such contexts. Hence, we speculate that the observed effect on decision speed in this study could be the result of variation in processing speed.

Shoal structure and dynamics in general did not vary between the telencephalon size selection lines with the exception of differences in speed. An earlier comprehensive examination of shoaling structure and dynamics using the same guppy shoals as the current study, found that the small and large telencephalon size shoals did not differ in seven aspects of collective motion, including swimming speed, when exploring an open arena (Boussard et al. 2024). Together with those findings, we conclude that shoaling structure and dynamics in general are not considerably different between the telencephalon size selection lines. Transferring information on potential threats throughout a group relies on simple interaction rules between neighbor individuals (Couzin 2009; Couzin and Krause 2003), that most likely do not require additional neural tissue to be processed (Boussard et al. 2024; Corral-Lôpez et al. 2023; Kotrschal et al. 2018). Instead, variation in shoaling structure and dynamics are most likely influenced by factors such as variations in predator pressure (Herbert-Read et al. 2017; Huizinga et al. 2009; Ioannou et al. 2012; Magurran et al. 1992; Seghers 1974; Wood and Ackland 2011), plastic responses to shoal composition (Croft et al. 2003), or individual differences in sociability and boldness (Jolles et al. 2017). Furthermore, artificial selection on schooling propensity did not result in differences in telencephalon size between selection and control lines (Corral-Lôpez et al. 2023). Our results further strengthen the suggestion that the cognitive processing underlying shoaling dynamic is a more fundamental aspect of cognition, and that variation in telencephalon size is not a strong driver for shoaling dynamics in guppies. This also implies that since we found a difference between the telencephalon size selection lines in collective decision-making, predator avoidance is likely a social decision that stems from the more advanced cognitive processing by each individual, that then has to coordinate with the group to reach consensus.

It is possible that the faster collective decision-making in large telencephalon size guppy shoals is caused by swimming speed capacity and rather than faster cognitive processes. However, we believe that this is highly unlikely for at least two reasons. First, swimming speed did not differ between the telencephalon size selection lines in an open arena, using these guppy shoals (Boussard et al. 2024). Second, swimming speed is mainly controlled by neurons in the midbrain and not in the telencephalon (Uematsu et al. 2007). Since there are no differences between the telencephalon size selection lines in the brain regions that control swimming speed (Fong et al. 2021; Triki et al. 2023) it is unlikely that selection on relative telencephalon size cause differences in the proximate mechanism generate swimming speed. Hence, we propose that there are no general differences in swimming speed between the telencephalon size selection lines, and we conclude that the differences in swimming speed are most likely caused by the differences in decision-making latencies.

An important implication of our discovery is that it provides additional support for the mosaic brain evolution hypothesis, supported by the independent evolution of relative telencephalon size in guppies (Fong et al. 2021). The mosaic brain evolution hypothesis predicts that individual brain regions should evolve relatively independently from other brain regions (Barton and Harvey 2000; de Winter and Oxnard 2001; Striedter 2005). The large energetic cost to produce and maintain neural tissue may favor heritable allocation of resources into specific brain regions processing relevant sensory information from existing ecological challenges (Aiello and Wheeler 1995; Fong et al. 2021; Isler and van Schaik 2006; Isler and Schaik 2009; Striedter 2005). Since brain tissue is costly, other organs or energetically costly biological events are expected to trade off with brain size (Isler and van Schaik 2006; Isler and Schaik 2009; Kotrschal et al. 2013). However, no trade offs between telencephalon size and either gut size or offspring reproduction have been reported in these selection lines (Fong et al. 2021; Triki et al. 2023). Yet, a larger telencephalon has been demonstrated to be advantageous in several standard cognitive tests (Triki et al. 2023). Together with the faster collective decision-making to avoid the model predator, this supports that mosaic brain evolution can be an efficient driver of cognitive evolution.

To summarize, we conclude that collective decision-making speed under predator threat is improved in guppy shoals with larger telencephalon size. We propose that a larger telencephalon generates faster cognitive processing that accompany faster collective decisions. Our results highlight that evolutionarily induced mosaic variation in brain region size may be an important driver of individual fitness.

## Supplementary Information

Below is the link to the electronic supplementary material.

Supplementary Material 1

## Data Availability

No datasets were generated or analysed during the current study.
