# Supplementary material for: Collective decision-making under predator threat is faster in guppy shoals selected for larger telencephalon size
Source: Anim Cogn. 2025 Oct 21;28(1):82. doi: 10.1007/s10071-025-02003-7 (PMC12540551; doi:10.1007/s10071-025-02003-7)
Supplement: Supplementary file 1 — Supplementary Material 1 [file 10071_2025_2003_MOESM1_ESM.docx]

Supplementary material

**Table S1.** We examined the effect of telencephalon size on collective decision-making latency and accuracy, and time to leave the release zone in the start arm. Reported are **A**) Harzard ratio (HR) ± 95% CI and p-values obtained from a Cox regression assessing collective decision-making latency assessing collective decision-making mean latency, **B**) Chi-square values (χ^2^), degree of freedom (d.f.) and p-values obtained from a generalized linear model (GLM) assessing mean collective decision-making accuracy, **C**) χ^2^-values, d.f. and p-values obtained from a GLM assessing time to leave start zone, and **D**) F-values, d.f. and p-values from linear models (LM) assessing shoaling structure and dynamics.

**A**)

| Latency (s), Cox regression | | | |
| --- | --- | --- | --- |
|  | HR ± 95% CI |  | p-value |
| Telencephalon size | 1.70 (1.17, 2.45) |  | 0.005 |
| Replicate 2 | 1.56 (1.00, 2.45) |  | 0.05 |
| Replicate 3 | 0.69 (0.44, 1.07) |  | 0.10 |

**B**)

| Accuracy, GLM |  |  |  |
| --- | --- | --- | --- |
|  | χ^2^ | d.f. | p-value |
| Telencephalon size | 2.07 | 1 | 0.15 |
| Replicate | 3.02 | 2 | 0.22 |

**C**)

| Time to leave release zone, GLM |  |  |  |
| --- | --- | --- | --- |
|  | χ^2^ | d.f. | p-value |
| Telencephalon size | 1.35 | 1 | 0.24 |
| Replicate | 2.25 | 2 | 0.32 |

**D**)

| Main sub-shoal size, LM | | | |
| --- | --- | --- | --- |
|  | F-value | d.f. | p-value |
| Telencephalon size | 1.20 | 1 | 0.28 |
| Replicate | 2.71 | 2 | 0.07 |
| Body size | 6.59 | 1 | 0.01 |
| Alignment score, LM |  |  |  |
| Telencephalon size | 0.56 | 1 | 0.45 |
| Replicate | 0.001 | 2 | 0.99 |
| Body size | 4.10 | 1 | 0.05 |
| Speed | 32.42 | 1 | < 0.001 |
| Attraction, LM |  |  |  |
| Telencephalon size | 1.38 | 1 | 0.24 |
| Replicate | 1.11 | 2 | 0.33 |
| Body size | 31.91 | 1 | < 0.001 |
| Speed, LM |  |  |  |
| Telencephalon size | 7.14 | 1 | 0.009 |
| Replicate | 7.46 | 2 | < 0.001 |
| Body size | 21.03 | 1 | < 0.001 |

**Table S2.** We examined the relationship between telencephalon size, sex and their interaction in collective decision-making latency and accuracy, and time to leave the release zone in the start arm. Reported are **A**) Harzard ratio (HR) ± 95% CI and p-values obtained from a Cox regression assessing collective decision-making latency, **B**) Chi-square values (χ^2^), degrees of freedom (d.f.) and p-values obtained from a generalized linear model (GLM) assessing collective decision-making accuracy. **C**) χ^2^-values, d.f. and p-values obtained from a GLM assessing time to leave the release zone.

**A**)

| Latency (s), Cox regression | | |
| --- | --- | --- |
|  | HR ± 95% CI | p-value |
| Telencephalon size | 1.79 (1.24, 2.60) | 0.002 |
| Sex | 1.96 (1.34, 2.86) | < 0.001 |
| Replicate 2 | 1.70 (1.08, 2.68) | 0.02 |
| Replicate 3 | 1.72 (0.46, 1.12) | 0.14 |
| Telencephalon × sex | 1.05 (0.50, 2.17) | 0.90 |

**B**)

| Accuracy, GLM | | | |
| --- | --- | --- | --- |
|  | χ^2^ | d.f. | p-value |
| Telencephalon size | 2.34 | 1 | 0.13 |
| Sex | 38.88 | 1 | < 0.001 |
| Replicate | 2.99 | 2 | 0.22 |
| Telencephalon × sex | 0.16 | 1 | 0.69 |

**C**)

| Time to leave release zone, GLM | | | |
| --- | --- | --- | --- |
|  | χ^2^ | d.f. | p-value |
| Telencephalon size | 1.57 | 1 | 0.21 |
| Sex | 11.41 | 1 | < 0.001 |
| Replicate | 2.47 | 2 | 0.29 |
| Telencephalon × sex | 0.06 | 1 | 0.80 |

**Table S3.** We examined the relationship between telencephalon size, sex and their interaction on shoal structure and dynamics in guppy shoals. Reported are F-values, degrees of freedom (d.f.) and p-values from linear models (LM). Reported is also the covariates replicate, body size and speed when controlling for activity.

| Main sub-shoal size, LM | | | |
| --- | --- | --- | --- |
|  | F-value | d.f. | p-value |
| Telencephalon size | 1.06 | 1 | 0.30 |
| Sex | 0.88 | 1 | 0.35 |
| Replicate | 2.53 | 2 | 0.08 |
| Body size | 0.50 | 1 | 0.48 |
| Telencephalon size × sex | < 0.01 | 1 | 0.94 |
| Alignment score, LM |  |  |  |
| Telencephalon size | 0.56 | 1 | 0.46 |
| Sex | 0.81 | 1 | 0.37 |
| Replicate | < 0.01 | 2 | 0.99 |
| Body size | 0.22 | 1 | 0.64 |
| Speed | 26.83 | 1 | < 0.001 |
| Telencephalon size × sex | 1.77 | 1 | 0.19 |
| Attraction, LM |  |  |  |
| Telencephalon size | 1.69 | 1 | 0.20 |
| Sex | 2.78 | 1 | 0.10 |
| Replicate | 1.83 | 2 | 0.17 |
| Body size | 3.82 | 1 | 0.05 |
| Telencephalon size × sex | 1.83 | 1 | 0.49 |
| Speed, LM |  |  |  |
| Telencephalon size | 0.15 | 1 | 0.70 |
| Sex | 3.51 | 1 | 0.06 |
| Replicate | 4.61 | 2 | 0.01 |
| Body size | < 0.001 | 1 | 0.98 |
| Telencephalon size × sex | 4.03 | 1 | 0.047 |
